# A Dual-Layer Weight-Leader-Vicsek Model for Multi-AGV Path Planning in Warehouse

**DOI:** 10.3390/biomimetics8070549

**Published:** 2023-11-15

**Authors:** Shiwei Lin, Ang Liu, Jianguo Wang

**Affiliations:** Faculty of Engineering and Information Technology, University of Technology Sydney, Sydney, NSW 2007, Australia; shiwei.lin-1@student.uts.edu.au (S.L.); ang.liu@student.uts.edu.au (A.L.)

**Keywords:** path planning, Vicsek model, leader–follower, hybrid A*, multiple automatic guided vehicles

## Abstract

Multiple automatic guided vehicles are widely involved in industrial intelligence. Path planning is crucial for their successful application. However, achieving robust and efficient path planning of multiple automatic guided vehicles for real-time implementation is challenging. In this paper, we propose a two-layer strategy for multi-vehicle path planning. The approach aims to provide fast computation and operation efficiency for implementation. The start–destination matrix groups all the vehicles, generating a dynamic virtual leader for each group. In the first layer, the hybrid A* algorithm is employed for the path planning of the virtual leaders. The second layer is named leader–follower; the proposed Weight-Leader-Vicsek model is applied to navigate the vehicles following their virtual leaders. The proposed method can reduce computational load and achieve real-time navigation by quickly updating the grouped vehicles’ status. Collision and deadlock avoidance is also conducted in this model. Vehicles in different groups are treated as dynamic obstacles. We validated the method by conducted simulations through MATLAB to verify its path-planning functionality and experimented with a localization sensor.

## 1. Introduction

With the development of robotic technologies, mobile robots are implemented in commerce and industry. Automated Guided Vehicles (AGVs) enhance transportation efficiency with less cost [1,2]. They are utilized in the industry as a part of industrial intelligence, intelligent logistics, and intelligent factories [2,3]. AGVs comprise the industry’s unit load vehicles, towing vehicles, forklifts, and pallet trucks [4]. AGVs are applied to diminish labour costs and improve safety for the high demands in a production environment [5,6]. Servo handling systems, warehousing systems, logistics, and storage industries employ AGVs in various areas [6,7], including transportation, transhipment, distribution, and material handing in manufacturing [8].

AGVs can transfer products at high speeds in a chaotic situation. They are implemented in production lines with flexible development in modern manufacturing, incorporating automated intelligent control systems [4,6,9]. With sensors’ help, detecting obstacles and automatically eliminating problems ensure the intelligence and adaptability of AGVs [6]. AGV navigation in the industrial environment usually implements the fixed line or the coupled approach, which adapts the single-robot navigation methods. It lacks the flexibility and the possibility of real-time implementation. It has highly demanded that AGV have a quick and flexible route setting and adapt to the dynamic environment. The motivation of this study is to provide fast computed path planning with flexibility and scalability for multi-AGV systems, addressing most situations.

This paper introduces a new algorithm for this purpose. Its main contribution is as follows:

- A novel path-planning approach for multi-AGV systems based on the improved Vicsek model with a leader–follower structure. The bio-inspired approaches are widely used in multi-robot path planning. The Vicsek model is adapted for this case because the AGVs aim to move as a swarm operation and provide quick computation for the entire system.
- Offering the fast path setting for multiple AGVs in one calculation step, which differs from the other path-planning algorithms that repeat the algorithms for every robot.
- For real-time implementation, it provides faster computation and adaption to the environment.

Instead of navigating all the AGVs directly, a set of virtual leaders is introduced in the AGV swarms to navigate all the AGVs, combining search and intelligent algorithm advantages. Biological patterns and the leader’s principle are the main concepts in this model, and it integrates the coupled and decoupled approach. The hybrid centralized decentralized is proposed for determining the leader’spath while providing flexibility for AGVs. Each AGV follower collects data from its neighbours and is led by the virtual leaders without the restriction of the current group. One of the significant advantages of the proposed multi-AGV path-planning system is that it requires less computational load for real-time implementation.

The Leader-Vicsek model was published in [10], and this paper is an extension. The published paper introduces the concept of the novel Weight-Leader-Vicsek model, but it uses simple, straightforward path planning for the leader and traditional Vicsek update equations for followers. Nevertheless, this paper uses a dual layer to improve the model as an enhanced Weight-Leader-Vicsek model (WLVM). It generates the dynamic virtual leader by the hybrid A* algorithm and uses a start–destination matrix to determine the swarms’ integration, and collision avoidance is achieved by priority. Also, the followers’ position and angle updates are improved by adding the weight for the average angle and considering the current leader’s angle. The simulations are conducted in a new scenario, and WLVM is compared with other algorithms.

This paper offers a multi-AGV path-planning approach for optimizing automatic transportation in commercial or industrial warehouses. The paper is organized as follows. Section 2 reviews path-planning algorithms and the multi-AGV navigation approaches. Weight-Leader-Vicsek model is proposed in Section 3 for multi-AGV path planning and navigation. Experiment results are demonstrated in Section 4 to validate the approach, and the conclusion is in Section 5.

## 2. Related Work

The Automatic Guided Vehicle (AGV) plays a crucial role in the intelligent transportation system. AGVs are the primary automated equipment that carry materials and process unmanned distribution and sorting in an unmanned storage environment [11]. Path planning has been the most crucial consideration of mobile robot navigation, which plans the path from the start to the target for mobile robots [3].

Obstacle avoidance functions must be developed to operate AGVs when considering dynamical limitations and dynamical safety [1,12]. Meta-heuristic-based methods [13], graph search-based methods [2,14], mathematical optimization-based methods [1], and potential field and navigation-based methods [15] are the four main categories for navigation algorithms.

Additionally, adapting the classic graph search algorithm is implemented widely for AGV path planning, such as the A* and Dijkstra [16]. The limitation of the improved A* algorithm is that the path cannot be guaranteed optimal and is conducted under simulation. Different algorithms can be combined; for example, the Dijkstra algorithm is for initial static path planning, and the virtual potential function algorithm is for dynamic path planning [17]. The drawback of this study is that static or dynamic obstacles have yet to be tested in real-life testing. Sampling-based methods are also significant for single AGV path planning, such as the Voronoi graph and rapidly exploring random trees (RRT) methods [18]. Future research on the improved RRT must combine obstacle avoidance and data-driven path planning.

The mathematical optimization-based approach also consists of open-loop and closed-loop strategies [1]. A nonlinear model predictive control (MPC) algorithm has been proposed for large-size AGVs with onboard LIDAR sensors and a 14 DoF vehicle dynamics model [1]. Artificial potential field (APF) methods [19] and the probabilistic road map [20] are also proposed for motion planning. The limitations of these studies include vehicle control, moving obstacles, and real-time implementation.

The AGV path-planning algorithms introduced above are mainly focused on individual AGVs. For the venues with multiple AGVs operated simultaneously, the dynamic environment due to other AGVs and people needs to be handled safely and efficiently.

Multi-AGV systems have become more popular because of their powerful task-solving complexity, continuous operation, reduced maintenance and operational costs, and broad convergence, flexibility, and versatility [21,22]. Multi-AGV optimization scheduling can improve the logistics transportation system structure and system operation efficiency and reduce transportation costs [23,24].

Finding the best path quickly and avoiding collision is worth studying in an AGV operation [25]. AGVs obtain the paths from the multi-AGV scheduling system, and they sense the surroundings independently and communicate with others by sending poses [26]. AGVs are usually guided by optical, electromagnetic, and laser navigation technologies or combinations of them, following the arranged path and avoiding collisions [27]. The research on multi-AGV routing can be classified as semi-dynamic, fully-dynamic, and static routing [28].

Vehicle dispatching, positioning, vehicle routing, scheduling, and collision and deadlock avoidance are considered for designing vehicle transportation systems [29]. Current multi-AGV systems commonly implement the centralized control architecture to perform tasks, such as motion coordination, mission allocation, and path planning [30]. The centralized control system is referred to as the warehouse management system (WMS) [31]. However, each AGV plans its path for the distributed approaches and resolves deadlocks or collisions by communicating with its neighbours. Vehicle autonomy and distributed computation are characteristics of decentralized methods [21].

Moreover, a decentralized approach for determining the shortest paths and motion coordination based on nonholonomic vehicle constraints is presented in [21]. It needs to consider the other vehicles’ motion plans or locations in future work. A regional control model is introduced for distributed control for the multi-AGV system in [32] to minimize the complexity of scheduling issues. The limitations are related to real manufacturing companies and distributed control mechanisms.

An ant-agent optimized by a repulsive potential field is developed to combine centralized and decentralized control and avoid path conflicts with stability and efficiency [28]. The approach’s limitations are that it cannot determine whether the method is optimal or considers the working condition of AGV. A multi-AGV path-planning method improves ant colony algorithms according to prioritized planning, considering battery management in [33] as a decentralized algorithm, whereas it only uses simulation to verify the results.

The metaheuristic algorithms are widely used for optimization problems for AGV systems, such as task allocation [24] and path planning [34]. Multi-agent path-planning algorithms can be divided into rule-based, search-based, and learning-based [35]. Rule-based algorithms use mature solutions for path planning by transferring the problem to other problems; search-based algorithms implement heuristic search algorithms and are classified as decoupled search, and coupled search algorithms [36], and learning-based algorithms obtain optimal solutions from suboptimal solutions [35].

A multi-AGV probabilistic time-constrained path-planning algorithm is also based on the A* heuristic algorithm with dynamic stochastic network theory in [22]. An improved A* path-planning algorithm is introduced for a grid-shaped network, ensuring the locating and execution of motion commands [25]. The unidirectional directed graph method combined with the A* algorithm for AGV path planning in a multi-AGV scheduling system is presented [37]. The limitations of these improved A* algorithms consist of the iterative update whichis not adaptive of path arc time consumption [22], lacking experiments [22,25,37], dynamic scheduling algorithms, or path optimality [37].

Heuristic information and elastic time window are considered in the improved ant colony algorithm [38], and the conflict resolution strategies are based on the priority of AGV task scheduling. The drawbacks of the algorithm include transportation equipment, actual operation, and path conflict. A hybrid genetic algorithm-particle swarm optimization is proposed for multi-AGV path planning with a fuzzy logic controller in [36], combining scheduling and path planning. The shortcoming focuses on dynamic real-time scheduling in a large-scale system. A genetic algorithm is improved to consider the highest charging utilization rate and the shortest path to plan the optimal path for multi-AGV, whereas it lacks real operation [11].

Deadlock avoidance is the primary consideration during multi-AGV path planning. Nodes describe the physical locations, whereas the grids are independent spaces in the environment [23]. The node-based coordination strategies strictly avoid the AGVs occupying a common node. The authors of [23] propose deadlock strategies by combining nodes and grids, and future work will be integrated with a distributed framework for scalability. A structural online control policy is proposed for multi-AGV deadlock resolution based on analyzing the system as discrete events [39]. The topological graph and roadmap work for the AGVs’ subsequent coordination by local negotiation and shared resources as a holistic approach in industrial warehouses [40]. The shortcomings focus on the task allocation mechanism and real factors of the graph weights.

However, the multi-agent algorithms plan the path independently and lack consideration of moving obstacles and real-time implementation. Most evolution-based and swarm-based algorithms are bio-inspired, and the biological pattern is considered when developing the new algorithm. This paper proposes a Weight-Leader-Vicsek model algorithm, which incorporates the advantages of decentralized and centralized approaches. Each AGV collects data from relevant AGVs, determines its path, and achieves collision avoidance. At the same time, a central decision-maker assigns the multi-AGV groups and virtual leaders for the defined groups. AGV control variables can be gained with faster computational speed and less complexity. This model provides path-planning functionality simultaneously for grouped AGVs and treats each group as different swarms.

## 3. WLVM with Virtual Leaders and Weight

### 3.1. Preliminary Knowledge: Vicsek Model

The Vicsek model is introduced for particles with biologically motivated interaction with self-ordered motion and is proposed in [41]. The Vicsek model can be expressed by (1) and (2), and the algorithm is shown in Algorithm 1. Biological subjects spin in the same direction and move as their neighbourhood for interaction in an L∗L square region [41,42]. For a multi-agent system, the Vicsek model has been improved by taking a fixed number of neighbours and a percentage of neighbours into account as the remote neighbours’ strategy [43]. The hierarchical Weighting Vicsek model is proposed for flocking navigation, and it assigns different layers for the drones with weights to enhance the convergence speed, analyzing the involved parameters [44].

(1) $$x_{i}\left(t+1\right)=x_{i}\left(t\right)+v_{i}\left(t\right)\Delta t$$

(2) $$\theta_{i}\left(t+1\right)={\langle \theta (t)\rangle }_{r}+\Delta \theta$$

where $x_{i}\left(t+1\right)$ is the location of the particle *i*, the velocity $v_{i}$ is gained through an absolute velocity, and the angle is represented by $\theta_{i}\left(t+1\right)$. ${\langle \theta (t)\rangle }_{r}$ represents the average angle of the neighbouring particles within a circle with radius *r*. Δθ denotes a random number from $\left[-\frac{\eta }{2},\frac{\eta }{2}\right]$ as noise.
**Algorithm 1:** Vicsek model**1**
Initialize parameters**2****for** time←1 to $time_{max}$
**do****3**


Calculate averageTheta**4**

x←x+vel∗cos(theta)∗dt**5**

y←y+vel∗sin(theta)∗dt**6**

theta←averageTheta+noise**7****end**

### 3.2. Problem Statement

**Figure 1:**
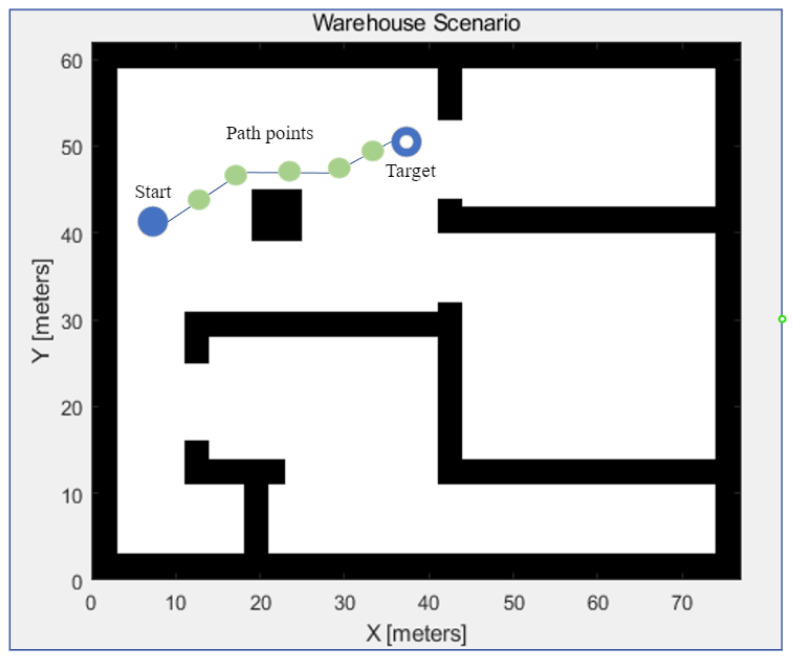
Path planning in the map.

The basic components of AGV path planning include the start, the target, and the environment. Path planning aims to generate the path from the start to the target without collisions. Figure 1 indicates an example of AGV path planning. As the occupancy map, the map uses the binary number to represent the locations in a 2D space. It can be transformed into a grid map or nodes for performing the heuristic or other path-planning algorithms. The obstacles or the walls are annotated in black.

The AGV is supposed to move from the start to the target, and green points demonstrate the path points. The obstacles or walls are treated as static obstacles, and the generated path should not be overlapped with the static obstacles. The path points are represented by $r_{1}^{k},r_{2}^{k},\ldots ,r_{n}^{k}$. *r* is the position of the current iteration *k* for *n*th AGV, and $r_{n}^{k}$ is $\left(x_{n}^{k},y_{n}^{k}\right)$.

Collision and deadlock avoidance are necessary for the multi-AGV system. Each AGV is assumed to communicate with other AGVs treated as dynamic obstacles. The deadlock of AGVs should be avoided for great system performance, and the adjusting progress is based on priority. The adjusted position of AGV *n* is represented by $r_{n-new}$.

### 3.3. Algorithm Description

#### 3.3.1. Overview

WLVM is proposed for a multi-AGV path-planning algorithm to improve the Vicsek model because the Vicsek model cannot achieve practical path planning in the industry. WLVM assigns the virtual leaders to collaborate with the grouped AGVs and guide the followers to reach their destinations, considering collision avoidance.

**Figure 2:**
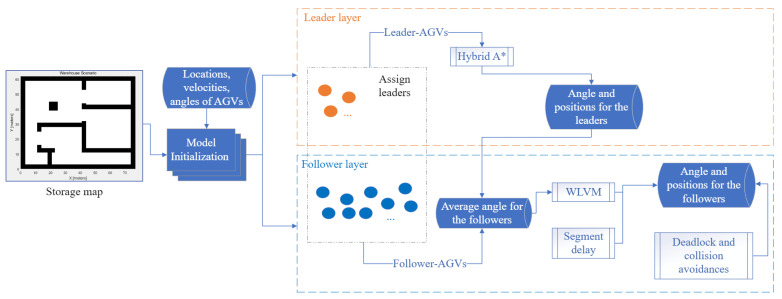
WLVM process.

Figure 2 describes the process of WLVM. According to a real industrial environment, the storage map is generated. Points represent the map; 0 is open space for movement, whereas 1 represents the wall or obstacles. The number of AGVs, velocities, angles, and locations are set for model initialization. The AGVs are divided into swarms based on their locations and destinations with assigned virtual leaders. The positions and angles of virtual leaders are computed by the hybrid A* algorithm. The follower-AGVs use the status of the leader in the current group to obtain the average angle within the defined path for WLVM. The AGVs implement a segment delay function to be separated by a certain distance for optimal arrangements. The AGVs avoid vehicle congestion and deadlock. Ref. [10] presents the design of the model for the AGV system.

Table 1 saves the positions and angles of each swarm during the path-planning process. *N* stands for the number of the AGVs, and Virtual Leader represents the leader in the group. WLVM saves the positions and angles for follower-AGVs and virtual leaders in each iteration and is used for further updating.

#### 3.3.2. Dynamic Virtual Leader

**Figure 3:**
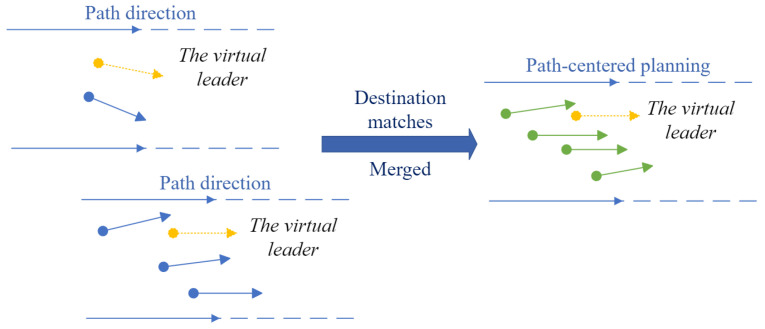
Principle of WLVM.

Dynamic virtual leaders are implemented in WLVM for shorter paths, faster convergence, and more accurate direction for planning the path to arrive at the destination. Each multi-AGV group has one virtual leader. Figure 3 demonstrates the principle of the dynamic virtual leader. When a new AGV joins the current group, the AGVs of the multi-AGV group will treat it as part of the current group, and the group dynamically generates the virtual leader. The virtual leaders are generated in a static environment. When the AGVs aim for different areas, the start–destination matrix makes the separation, which refers to Section 3.3.3.

The positions for each AGV are calculated by (3). The equations for calculating the angles for follower AGVs are as (4).

(3) $$\left(\begin{array}{c} x_{i}^{k+1}\\ y_{i}^{k+1} \end{array}\right)=\left(\begin{array}{c} x_{i}^{k}\\ y_{i}^{k} \end{array}\right)+v\Delta t\cdot \left(\begin{array}{c} cos\theta_{i}^{k}\\ sin\theta_{i}^{k} \end{array}\right)$$

(4) $$\theta_{i}^{k+1}=\omega_{1}\cdot arctan\frac{{\langle sin(\theta_{i}^{k})\rangle }_{p}}{{\langle cos(\theta_{i}^{k})\rangle }_{p}}+\omega_{2}\cdot \theta_{l}^{k+1}+\eta_{i}^{k}$$

where the average direction $arctan\frac{{\langle sin(\theta_{i}^{k})\rangle }_{p}}{{\langle cos(\theta_{i}^{k})\rangle }_{p}}$ is estimated along the path *p*, and the travel distance is represented by vΔt. $\eta_{i}^{k}$ denotes the noise. $w_{1}$ is a random number in (0,1), and the sum of $w_{1}$ and $w_{2}$ is 1. The swarm only considers the AGVs in the defined path in the same direction. The hybrid A* algorithm is integrated to calculate the position and angles for virtual leaders, and $\theta_{l}^{k+1}$ denotes the angle of the leader in the current iteration. It is adapted for multi-task implementation for virtual leaders.

When an AGV joins a new group, the virtual leader of that group will change if the AGV’s destination aligns with the group’s destination. If not, the group’s structure and leader remain unchanged. The leaders are predefined, while the followers keep gaining statuses from the neighbourhoods and then updating their status. It provides the possibility of real-time implementation.

For virtual leaders, angles and positions are generated by the hybrid A* algorithm, and the pseudo-code is indicated in Algorithm 2. A* is a widely used graph traversal algorithm because of optimal efficiency and completeness [45]. The hybrid A* algorithm is proposed in [46], which guarantees kinematic feasibility and continuous nature [47]. The heuristics are the maximum non-holonomic-without-obstacles and obstacle map, ignoring the nonholonomic nature [46]. The MATLAB navigation toolbox has the function for the hybrid A* algorithm.
**Algorithm 2:** Hybrid A***1**
Initialization of openset, closeset**2**
openset.push(start)**3****while** *openset is not empty* **do****4**

$x_{current}\leftarrow openset.popMinCostNode$**5**

**if** *exists RS path* **then****6**




return the path**7**

**end****8**

**for** $x_{next}$*calculated by kinematic equation* **do****9**



Collision avoidance**10**


**if** $x_{next}$ *not exists in closeset* **then****11**





$g\leftarrow g\left(x_{current}\right)+l\left(x_{current},x_{next}\right)$**12**





**if** $x_{next}$ *not exists in openset or*$g<g(x_{next})$**then****13**




$g(x_{next})\leftarrow g$**14**





$h\left(x_{next}\right)\leftarrow Heuristic\left(x_{next},x_{goal}\right)$**15**






$Pred\left(x_{next}\right)\leftarrow x_{current}$**16**





**if** $x_{next}$ *not exists in openset* **then****17**








openset.push($x_{next}$)**18**





**else****19**








openset.update(next node)**20**






**end****21**



**end****22**



**end****23**

**end****24****end**

**Figure 4:**
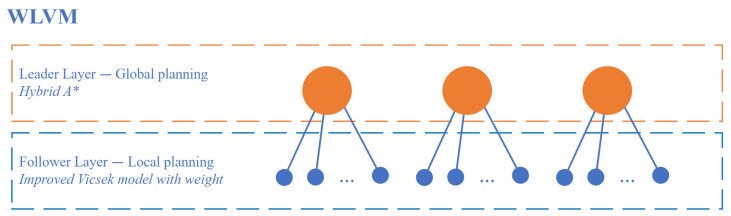
Dual layer of WLVM.

The hybrid A* and WLVM combination is shown in Figure 4, and the proposed WLVM is in Algorithm 3. The leaders are generated for each swarm, and they are unique. For the generated path for followers, the path needs to be smooth by the Spline curve for AGVs to operate. The leaders will be regenerated if the environment or group formation changes. The statuses of AGVs are defined as (5). The statuses of AGVs are dynamically assigned based on their roles in the current swarm. Once the AGV arrives at the destination, the status is −1.

(5) $$status=\left\{\begin{array}{c} 1,\mathrm{if}\;leader\\ 0,\mathrm{if}\;follower\\ -1,\mathrm{if}\;arrives \end{array}\right.$$

**Algorithm 3:** WLVM.
**Data**: x, y, leaderStart, leaderTarget
**1**

Initialize parameters

//
(n - the number of particles

**2**


n←size(x,2)+1


**3**


dt←1


**4**


change←1


**5**


$x\leftarrow \left[\begin{array}{cc} x & leaderStart_{x} \end{array}\right]$


**6**


$y\leftarrow \left[\begin{array}{cc} y & leaderStart_{y} \end{array}\right]$


**7**


theta←zeros(1,n)


**8**
**for** i←1 *to n* **do**
**9**




$theta\left(i\right)\leftarrow leaderStart_{theta}$


**10**

**end**

**11**
**while** *change* > 0 **do**
**12**




change←change−1


**13**


**for** *each changed swarm* **do**





//
(getting the positions and angles for the virtual leader

**14**






[LeaderPos,LeaderAngle]←HybridAstar


**15**




**for** time←1 to $time_{max}$ **do**
**16**







Calculate averageTheta 
**17**








x←x+vel∗cos(theta)∗dt


**18**








y←y+vel∗sin(theta)∗dt


**19**








theta←w1∗averageTheta+w2∗leaderAngle(time)+noise


**20**







Smoothen path
**21**







Deadlock and collision avoidance
**22**





**end**

**23**





Segment delay
**24**



**end**

**25**



Swarm evaluation
**26**


**if** *swarm changes* **then**
**27**






change←change+1


**28**



**end**

**29**

**end**


#### 3.3.3. Start–Destination

**Figure 5:**
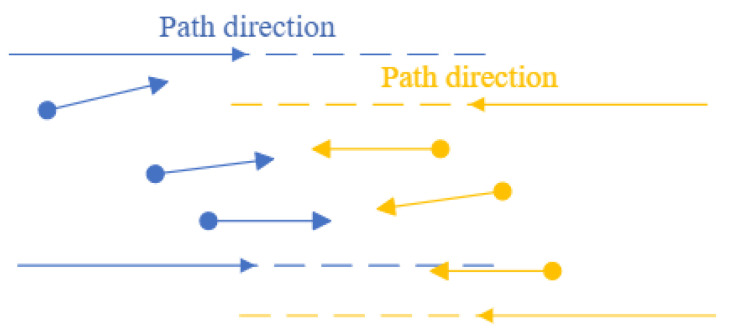
Groups with a different direction.

WLVM can handle the multi-agent motion directed to different areas, as shown in Figure 5. The start–destination matrix is saved in Table 2. It assigns the multi-AGV groups, determines each group’s destination, treats them as other swarms, and does not integrate them. *M* stands for the number of leader-AGVs, which is much less than the number of follower-AGVs. Start and Destination represent each leader’s origin and destination locations.

Keep updating positions and directions within the defined group based on the destination flag. When AGVs are in operation, they only consider the AGVs along the path in the same direction. Even if the other path with a different direction is closer, the AGVs would not be generated. It only concerns the AGVs on the current path.

#### 3.3.4. Segment Delay

**Figure 6:**
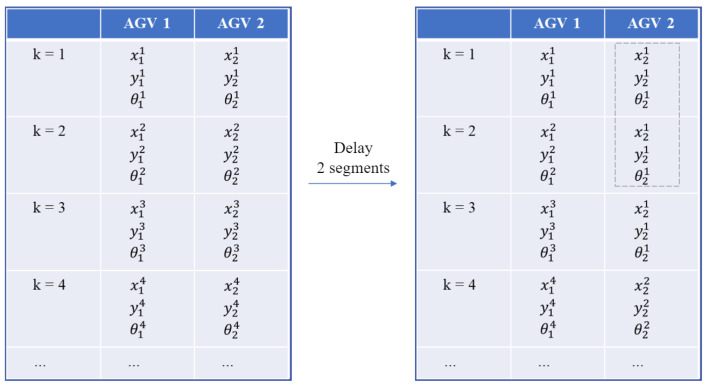
The positions after setting segment delay as two segments.

The proposed WLVM enables AGVs to travel as a swarm and reach the target, which results in inefficiency for loading and unloading, so segment delay is introduced to solve this problem. Segment delay allows for the spacing of AGVs to avoid them arriving simultaneously. This facilitates organized loading and unloading operations. Figure 6 indicates the operation of segment delay, and it sets segment delay as two segments. Each AGV follows the defined path with a different segment delay for departure in each swarm. The segment delay is set as five segments in the computational experiments to provide the buffer area among AGVs.

#### 3.3.5. Collision Avoidance

##### Obstacle Avoidance

Collision avoidance is necessary during the updating iterations to keep AGVs safe. There usually are some obstacles on the map in the practical implementation, so collision avoidance with the obstacles should be achieved. Collision avoidance of the virtual leaders is achieved by the hybrid A* algorithm. The follower-AGV utilizes the leader angle in the next iteration to determine the direction of the movement.

**Figure 7:**
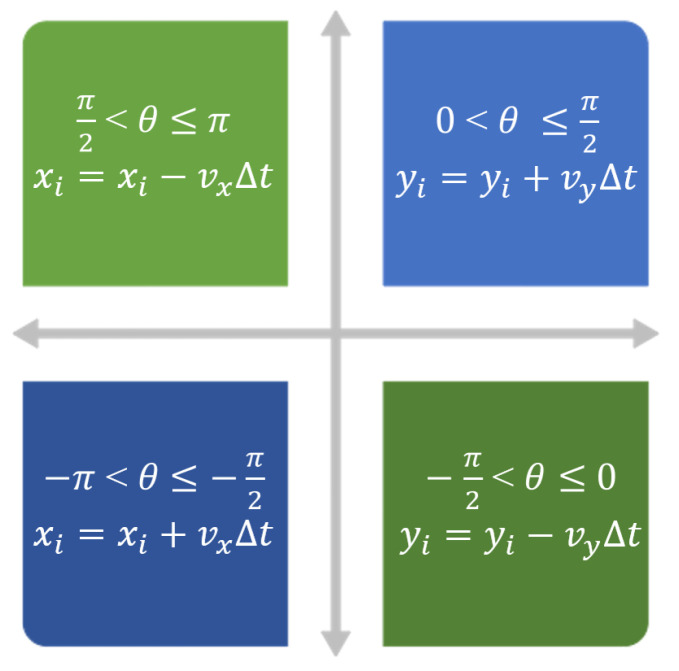
The AGV’s new position after collision avoidance.

Figure 7 demonstrates the movement of the AGV. Each obstacle or wall sets the buffer area as 1m. The leader angle θ indicates the movement of the path, and AGV is represented by *i*. If a path point represented by $r_{i}\left(x_{i},y_{i}\right)$ overlaps with the buffer area or the restricted area as the obstacles, it requires adjusting positions. The steps for achieving obstacle avoidance are as follows.

First, comparing θ with 0. If θ≥0 means the path is aimed to move upper/forward, then $y_{i}=y_{i}+v_{y}\Delta t$. Although θ<0 means the path moves lower, then $y_{i}=y_{i}-v_{y}\Delta t$. The AGVs follow the dotted line to change the Y locations. Second, comparing θ with $\frac{\pi }{2}$ or $-\frac{\pi }{2}$. If $\theta \geq \frac{\pi }{2}$ means the path is aimed to move left, then $x_{i}=x_{i}-v_{x}\Delta t$. Although $\theta \geq -\frac{\pi }{2}$, which means the path is moving right, then $x_{i}=x_{i}+v_{x}\Delta t$. The AGVs follow the dotted line to change the X locations.

##### Deadlock Avoidance

**Figure 8:**
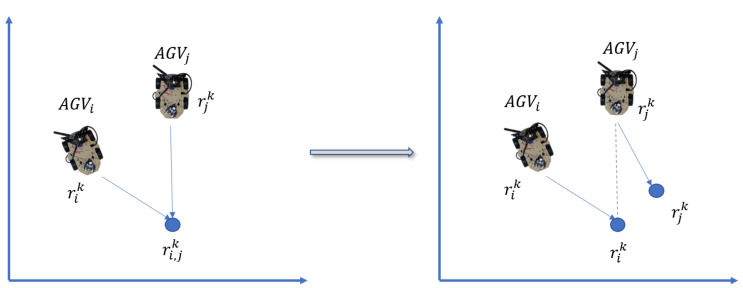
The AGV’s new position after deadlock avoidance.

The other AGVs in the predictable path or the moving obstacles are treated as dynamic obstacles. As shown in Figure 8, vehicle congestion must be avoided. The target location reserves only one vehicle in each iteration to avoid deadlock. The strategies to deal with the moving obstacles, except for other AGVs, refer to the previous section.

Deadlock avoidance involves priorities for AGVs, and high priority is assigned when AGVs carry goods close to the destination or have urgent tasks. The AGV with higher priority remains following the predicted path, whereas the other AGV moves to an available position. The priority is calculated based on (6), and the new position is gained by (7). The new position is updated in the robot’s path after ensuring it is available.

(6) $$priority_{i}=\omega_{1}\cdot priority_{task}+\omega_{2}\cdot distance$$

(7) $$r_{i-new}^{k+1}=r_{i}^{k+1}+rand$$

where *i* stands for the current robot, and distance is the distance from the current position to the destination. $priority_{task}$ represents the priority of each assigned task, and if the task is more urgent than others, $priority_{task}$ is higher. $w_{1}$ and $w_{2}$ are the weight of each factor, the sum of $w_{1}$ and $w_{2}$ is 1. $r_{i-new}$ stands for a new position of the robot *i*.

### 3.4. Comparison

**Figure 9:**
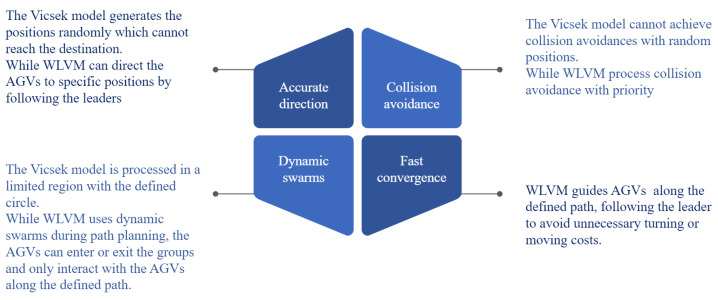
Comparison of Vicsek model and WLVM.

The proposed WLVM implements dynamic swarms and virtual leaders to ensure accurate direction and faster convergence, considering collision avoidance, the start–destination matrix to distinguish the destinations of swarms and the applications of multi-objective algorithms in the industrial environment. Figure 9 shows the advanced functionality of the WLVM. It also provides flexibility because of the dynamic swarms involved, and the follower-AGV can join or disconnect from the current group. The AGVs are assumed to exchange information during operations; if one AGV enters another area, it becomes a member of the new group.

The WLVM is novel for multi-AGV navigation, and it adapts the biological pattern because it achieves the path planning of several AGVs in one step. The traditional Vicsek model can describe the multi-agent movement, which is enhanced to improve WLVM. Following the biological pattern, AGVs move automatically as their neighbours do in the same direction if operating the same task. It is typical to involve several AGVs for one task; the improved WLVM achieves fast multi-AGV path planning by updating the positions and angles. It only requires calculation for the virtual leaders in the leader layer and one calculation step in the follower layer. Even though the number of AGVs is large, it obtains the path with a quick calculation.

## 4. Computational Experiments

### 4.1. Experiment Settings

**Figure 10:**
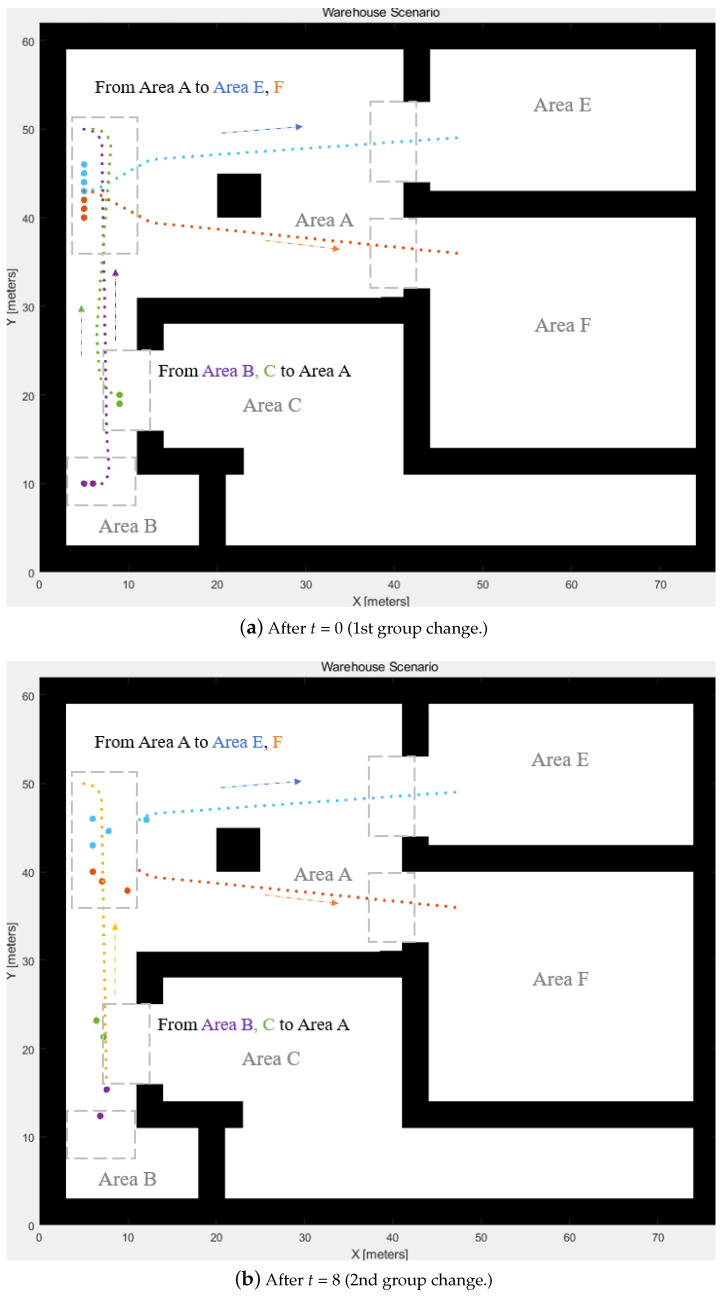
The path for virtual leaders.

Figure 10 generates the warehouse map and denotes the initial locations of AGVs; different colours indicate the different swarms. WLVM is validated through MATLAB. The start–destination matrix separates the swarms. Each delivery group is operating in Area A.

The multi-AGV system is engaged for deliveries from Area A because the three-dimensional storage system is implemented in the primary storage rooms: Area B to Area F. The materials are placed on the platform to transfer to the defined location by the pallet in the three-dimensional storage system.

Assumptions in the simulation are as follows:

- 7 AGVs depart from Area A to different storage areas for processing tasks, and 4 AGVs move to Area A for parking;
- Set segment delay for five segments in each swarm;
- AGVs with an absolute velocity of 1 m/s;
- Each AGV is equipped with a board that can operate WLVM;
- Each AGV has onboard sensors for localization and obstacle detection;
- Each AGV communicates with other AGVs, sending its positions, angles and statuses.

The groups’ settings are listed in Table 3.

### 4.2. Results

#### 4.2.1. Simulation

Figure 10 displays the path for the virtual leaders generated by the hybrid A* algorithm after the group changes. The directions are indicated along with the path. It also outlines the path area for each swarm. The blue path is from Area A to Area E, and the orange path is from Area A to Area F. The purple path is from Area B to Area A, and the green path is from Area C to Area A. Groups 1 and 2 from Area A are separated based on the simulation’s destination. Groups 3 and 4 are merged into one group when they are close to each other and aim for the same destination.

**Figure 11:**
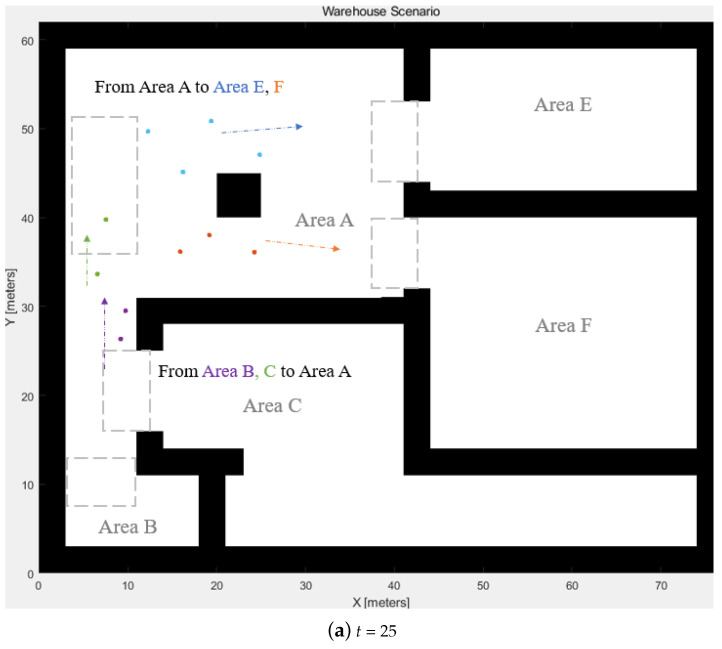

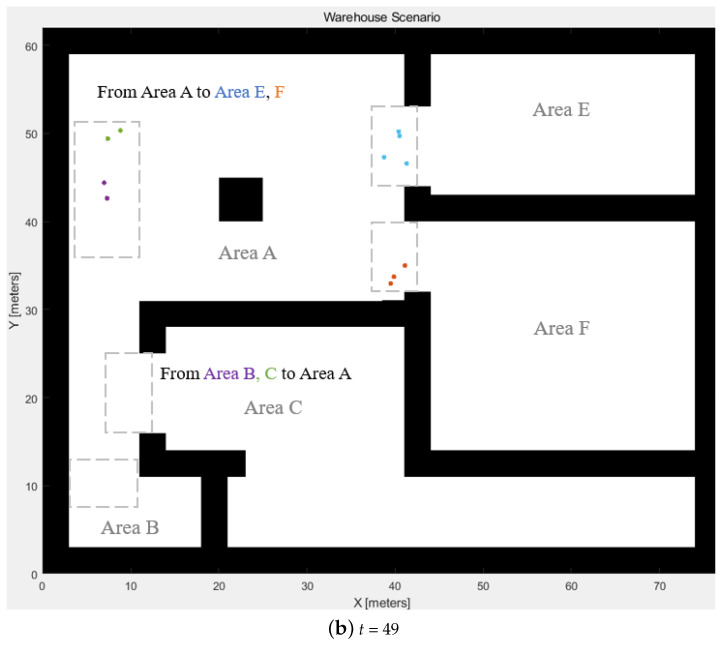
AGV positions during the path-planning process and the grey areas indicate the packing areas.

Figure 11 shows the AGVs’ paths. When two delivery groups pass the same area, collision avoidance ensures that AGVs operate safely. The start–destination matrix has played a role in distinguishing the multi-AGV group. The groups of inbound delivery are aimed at different main storage rooms with different virtual leaders. When the group arrives at the destination, the group is reminded of the current state, and the moving AGVs achieve collision avoidance based on the priorities.

The following Table 4 lists the performance measurements. The group number is assigned according to the respective swarms. The distance is the travelling distance for each AGV in the group, which refers to the distance each AGV must travel to accomplish its assigned task. The time is the consumed time for each swarm to complete the task.

#### 4.2.2. Experiment

The results of the simulation section are validated by the experiment with the Raspberry Pi robot and Ultra-Wide Band (UWB) for positioning. UWB provides centimetre-level positioning and high positioning accuracy in the indoor environment. The robot follows the designed path gained by the simulation.

**Figure 12:**
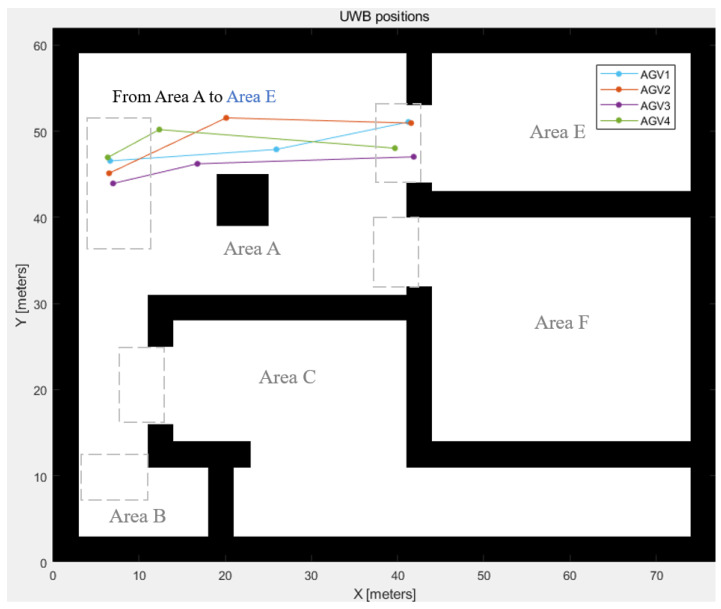
UWB positions following the generated path.

The experiment used the AGVs from Group 1, the inbound delivery group from Area A to Area E, with 4 AGVs. They followed the defined paths and gained positioning data from the positioning sensor. The positioning results were collected from the Decawave UWB sensors, and the robot carries the target for getting locations. The iterations of the results shown in Figure 12 are t=0, t=25, and t=49. The start positions are indicated as t=0, and the destinations are shown when t=49. The locations have some bias due to the sensor accuracy, which can be fixed by sensor fusion in future work.

### 4.3. Comparison

The proposed WLVM is compared with the RRT* algorithm [48] and an improved APF with deterministic annealing (DA-APF) [49] for path planning in Group 1, with a segment delay of five segments. The comparison of the runtime is listed in Table 5. RRT is a sampling-based algorithm that is popular for multi-constrained and high-dimensional path planning [50]. Sampling-based planners connect samples by constructing trees iteratively from the sampling distributions, and the planners deterministically or probabilistically draw random samples. APF uses attractive and repulsive potentials and treats the robot as an affected object. Wu et al. [49] apply a deterministic annealing strategy to improve the classical APF algorithm for path planning, changing the temperature parameters.

**Figure 13:**
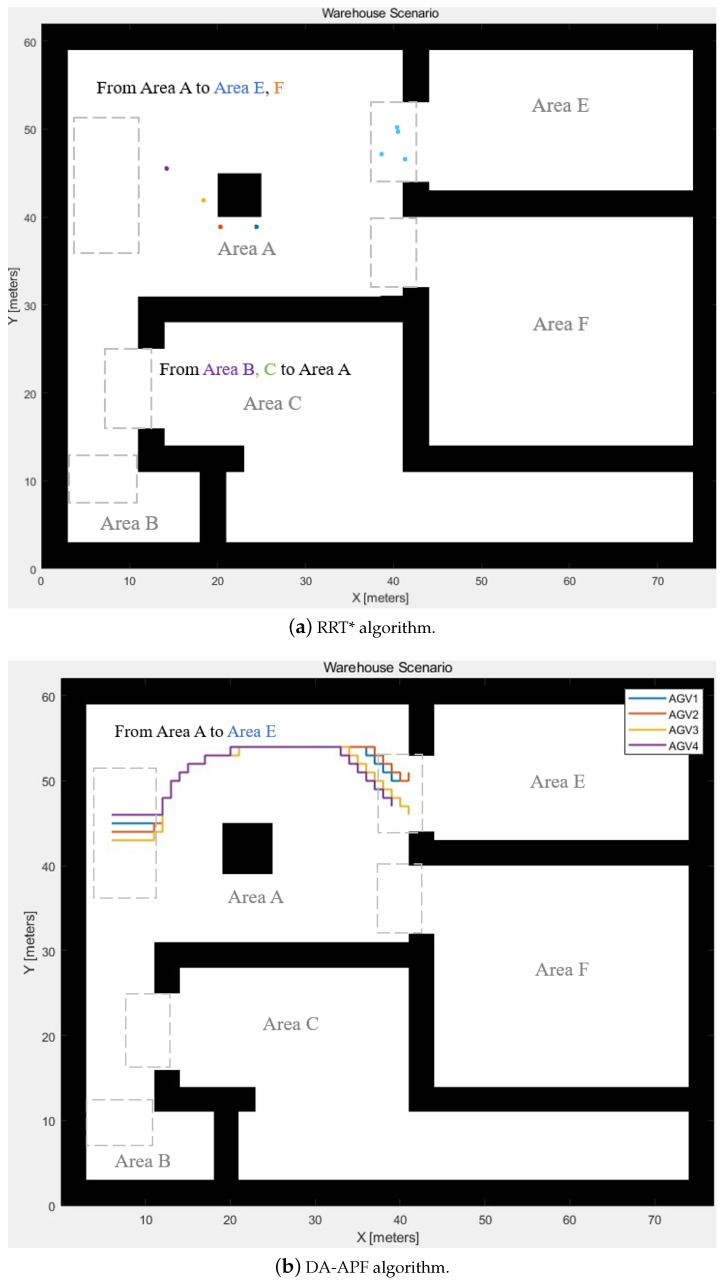
AGV positions generated by other algorithms.

The RRT* and DA-APF algorithms need to repeat the calculation for each AGV, whereas WLVM calculates the path for all AGVs at one step. WLVM provides fast path planning due to the computational speed. Figure 13 shows the AGV positions generated by the RRT* and DA-APF algorithms. Figure 13a shows the positions generated by the RRT* algorithm for *t* = 25 that are marked by different colours and the last positions when *t* = 64 that are marked by blue. If the number of AGVs dramatically rises, the computation time for the RRT* algorithm will rise rapidly, whereas it would not affect WLVM. Figure 13b indicates the path generated by the DA-APF algorithm. Although DA-APF achieves fast computation, it is still slower than WLVM for grouped AGVs.

## 5. Conclusions

The WLVM is proposed to improve the Vicsek model, and it develops dynamic virtual leaders and a start–destination matrix, considering the leaders’ direction with weight. Also, it is capable of providing scalability and flexibility for multi-AGV systems with fast and flexible path settings. The path-planning problem is formulated as a 2D space with the start and target locations, and it avoids static and dynamic obstacles. From the literature, most path-planning approaches plan the path independently. However, the proposed WLVM algorithm can offer the path settings in one calculation step for swarms.

The WLVM implements the virtual leader to navigate the follower-AGV in each multi-AGV group, and the leader’s positions and angels are generated by the hybrid A* algorithm. The proposed approach updates the statutes of AGVs with iterations, and the angles of AGVs consider the neighbour and the leader. Model and system initialization and multi-AGV group formation are completed through a centralized method, whereas it achieves the dynamic decentralized approach for each AGV.

It computes the follower-AGVs’ path with a quick computation, even though the number of AGVs is large. Unnecessary turning costs and path segments are avoided in this model. Each AGV only considers its neighbours in the path and tends to move as its neighbours. For swarm integration or separation, the start–destination matrix plays a role. It determines whether the multi-AGV group is aimed at the same destination and makes changes in the decentralized follower layer. Segment delay is implemented for optimal arrangement between AGVs for loading.

The proposed WLVM has the following benefits: accurate direction, dynamic swarms, fast convergence, and collision avoidance. It can achieve real-time implementation due to its computational speed and robust implementation. For the computational experiment, four groups with different settings demonstrate swarm separation and integration. The proposed algorithm is compared with other algorithms for path planning of four AGVs; the WLVM saves 98.39% and 47.41% computational time than the RRT* and DA-APF algorithms, respectively. WLVM is robust and simple for implementation during the AGVs’ or robots’ operations. The proposed algorithm can be applied to various scenarios involving the system of multiple robots, such as warehouses, logistics systems, ports, and airports.

The limitation of the proposed approach is that it does not consider the cost value during the path planning as the heuristic methodologies. Therefore, the generated path cannot be measured or estimated with the specific costs to determine whether the path is globally optimal. The robot’s dynamics and different speed scenarios must be considered during the real-world application. With the following considerations, WLVM would be more practical in the industrial environment for future work. Mission planning and task allocation can be included in the further improvement of this model. The multi-AGV system would be more practical if it involves fault tolerance during implementation. The combination of sensors and the sensor-fusion algorithm could be considered in further real experiments to estimate the angle and the position and detect and avoid obstacles. Implementing a neural network for the path-planning approach can be considered a potential improvement.

## Figures and Tables

**Table 1:** AGV navigation data in the same group.

Iteration	AGV 1	AGV 2	⋯	AGV n	Virtual Leader
1	$\left[\begin{array}{c} x_{1}^{1}\\ y_{1}^{1}\\ \theta_{1}^{1} \end{array}\right]$	$\left[\begin{array}{c} x_{2}^{1}\\ y_{2}^{1}\\ \theta_{2}^{1} \end{array}\right]$	⋯	$\left[\begin{array}{c} x_{n}^{1}\\ y_{n}^{1}\\ \theta_{n}^{1} \end{array}\right]$	$\left[\begin{array}{c} x_{L}^{1}\\ y_{L}^{1}\\ \theta_{L}^{1} \end{array}\right]$
2	$\left[\begin{array}{c} x_{1}^{2}\\ y_{1}^{2}\\ \theta_{1}^{2} \end{array}\right]$	$\left[\begin{array}{c} x_{2}^{2}\\ y_{2}^{2}\\ \theta_{2}^{2} \end{array}\right]$	⋯	$\left[\begin{array}{c} x_{n}^{2}\\ y_{n}^{2}\\ \theta_{n}^{2} \end{array}\right]$	$\left[\begin{array}{c} x_{L}^{2}\\ y_{L}^{2}\\ \theta_{L}^{2} \end{array}\right]$
⋮	⋮	⋮	⋮	⋮	⋮
*k*	$\left[\begin{array}{c} x_{1}^{k}\\ y_{1}^{k}\\ \theta_{1}^{k} \end{array}\right]$	$\left[\begin{array}{c} x_{2}^{k}\\ y_{2}^{k}\\ \theta_{2}^{k} \end{array}\right]$	⋯	$\left[\begin{array}{c} x_{n}^{k}\\ y_{n}^{k}\\ \theta_{n}^{k} \end{array}\right]$	$\left[\begin{array}{c} x_{L}^{k}\\ y_{L}^{k}\\ \theta_{L}^{k} \end{array}\right]$

**Table 2:** Start–destination matrix.

	Virtual Leaders			
**Location**	** $Leader_{1}$ **	** $Leader_{2}$ **	**⋯**	** $Leader_{M}$ **
Start	$Start_{1}$	$Start_{2}$	⋯	$Start_{M}$
Destination	$End_{1}$	$End_{2}$	⋯	$End_{M}$

**Table 3:** Group settings.

Group No.	Group	Priority	Number of AGVs	Color
1	Area A to E	1	4	Blue
2	Area A to F	2	3	Orange
3	Area B to A	2	2	Purple
4	Area C to A	3	2	Green

**Table 4:** Performance measurements.

Group No.	Group	Time	Distance	Group Changes
1	Area A to E	49 s	34.84, 36.46, 35.84, 34.02	Separated to Group 1 and 2
2	Area A to F	44 s	36.13, 37.36, 35.97	when *t* = 0
3	Area B to A	37 s	33.00, 34.21	Group 3 and 4 merged
4	Area C to A	35 s	30.82, 30.86	when *t* = 8

**Table 5:** Runtimes for the RRT*, DA-APF, and WLVM algorithms.

Algorithm	Runtime	Total Runtime
WLVM	Leader: 0.0757 s	0.0813 s
	AGV 1–4: 0.0056 s	
RRT*	AGV 1: 1.5522 s	5.0382 s
	AGV 2: 1.1777 s	
	AGV 3: 1.1550 s	
	AGV 4: 1.1533 s	
DA-APF	AGV 1: 0.0419 s	0.1546 s
	AGV 2: 0.0381 s	
	AGV 3: 0.0372 s	
	AGV 4: 0.0374 s	

## Data Availability

Data are contained within the article.

## Abbreviations

The following abbreviations are used in this manuscript:

AGV	Automatic guided vehicles
Multi-AGV	Multiple automatic guided vehicles
WLVM	Weight-Leader-Vicsek Model
RRT	Rapidly-exploring random trees
WMS	Warehouse management system
DA-APF	APF algorithm based on deterministic annealing
